# Correlation between ultrasonographic and cytologic features of thyroid nodules: a single-center cross-sectional study

**DOI:** 10.25122/jml-2024-0038

**Published:** 2024-06

**Authors:** Imane Ziani, Anouar Jamal, Imane Assarrar, Ikram Karabila, Siham Rouf, Hanane Latrech

**Affiliations:** 1 Department of Endocrinology-Diabetology and Nutrition, Mohammed VI University Hospital, Mohamed the First University, Oujda, Morocco; 2 Laboratory of Epidemiology, Clinical Research and Public Health, Mohamed the First University, Oujda, Morocco

**Keywords:** EU-TIRADS, fine-needle aspiration, malignancy, thyroid nodule, ultrasound, ATA, American Thyroid Association, CI, Confidence Interval, EU-TIRADS, European Thyroid Imaging Reporting and Data System, FNA, Fine Needle Aspiration, FNAC, Fine-Needle Aspiration Cytology, FT4, Tetraiodothyronine, OR, Odds Ratio, TBSRTC, The Bethesda System for Reporting Thyroid Cytopathology, TSH, Thyroid-Stimulating Hormone, TPOAb, Thyroid Peroxidase Antibody, TTW, Taller-Than-Wide, US, Ultrasound

## Abstract

A thyroid nodule is managed according to the clinical context, ultrasound (US) findings, and fine needle aspiration (FNA) results. Most thyroid nodules are benign; however, nodule classification is crucial to avoid unnecessary thyroid surgery. We conducted this study to compare the findings of fine-needle aspiration cytology (FNAC) expressed using the Bethesda system with the features of thyroid US classified using the EU-TIRADS classification to assess the risk of malignancy. A descriptive and analytical study involving 99 patients with thyroid nodules followed up in the Department of Endocrinology-Diabetology and Nutrition. Data were collected from medical records and analyzed using SPSS software V21. FNA was performed on 121 nodules using the BETHESDA system. These nodules were classified as malignant, suspicious for follicular neoplasm, and suspicious for malignancy in 5.8%, 5%, and 1.7% of cases, respectively. As for the EU-TIRADS 2017 classification, 59.5% of benign nodules were classified as EU-TIRADS III, whereas 66.7% of malignant nodules were classified as EU-TIRADS V and significantly related to malignant prediction (*P* = 0.000). The size of nodules was significantly correlated to the risk of malignancy (*P* = 0.013). Seventy-five percent of nodules with central vascularity were malignant (*P* = 0.012). Irregularity of nodule contours was significantly associated with the risk of malignancy, as 30% of nodules with irregular contours were Bethesda VI (*P* = 0.003). Hypoechogenicity was found in 77.8% of malignant nodules (*P* = 0.004). Additionally, only 9.2% of the nodules were taller than wide, of which 37.5% were malignant (*P* = 0.012). For a safe management strategy, US-guided FNAC should be performed on each suspicious thyroid nodule, given the correlation between EU-TIRADS classification features and the risk of malignancy.

## INTRODUCTION

A thyroid nodule, as defined by the American Thyroid Association (ATA), is a “discrete lesion within the thyroid gland that is radiologically distinct from surrounding thyroid parenchyma” [1]. Thyroid nodules are a frequently encountered reason for consultation. It is a common clinical finding, with a prevalence ranging from 2 to 6% for palpable nodules and 19 to 35% for ultrasound-detectable nodules [2]. The malignancy risk of thyroid nodules can be predicted by various clinical practice elements. They are managed based on the clinical context, ultrasound findings, and fine needle aspiration. When dealing with a thyroid nodule, the major issue is discerning its benign or malignant nature. Fortunately, 95% of thyroid nodules are benign, but 5% are malignant. Therefore, a treatment protocol needs to be established [3].

Ultrasound (US) is the reference imaging method for thyroid nodules. It has the advantage of being non-invasive and easily accessible. It is widely used in current practice for identifying and stratifying thyroid nodules, using multiple risk stratification systems, including the European Thyroid Imaging Reporting and Data System (EU-TIRADS) [4]. This EU-TIRADS is a scoring system that defines the risk of malignancy, thus determining whether the nodule requires a fine needle aspiration (FNA).

The FNA is a complementary technique to ultrasound that allows the cytological classification of thyroid nodules with a sensitivity of 95%[5]. They are mostly performed to diagnose and exclude malignant thyroid nodules. FNA findings are standardized by the Bethesda System for Reporting Thyroid Cytopathology (TBSRTC) [6]. This system reduces the rate of unnecessary thyroidectomies and helps plan the surgical modalities.

This study examined the diagnostic utility of thyroid ultrasound (US) in characterizing thyroid nodules and determining the necessity for FNA. It also aimed to study and compare ultrasound findings of thyroid nodules with cytological results and, more specifically, to describe and analyze the different ultrasound features predictive of malignancy (hypoechoic echogenicity, central and mixed vascularity, nodule size, irregular borders, and shape) and their correlation with cytological aspects.

## MATERIAL AND METHODS

### Study design and patients

**Figure 1:**
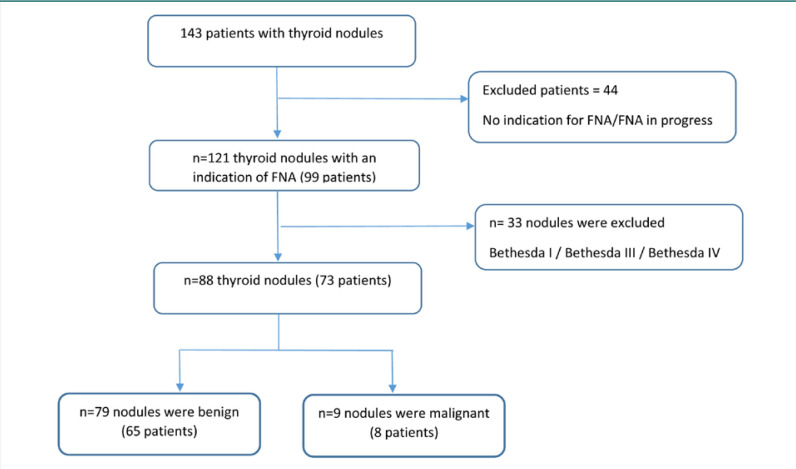
Flow chart showing the number (%) of patients with thyroid nodules in benign and malign categories (Bethesda V and Bethesda VI)

This study was a retrospective, descriptive, and analytical cross-sectional analysis conducted at the Endocrinology-Diabetology and Nutrtion Department of Mohammed VI University Hospital in Oujda, Morocco, over a three-year period. We included patients who presented with thyroid nodules, identified either incidentally, through self-examination, clinical examination, or due to thyroid-related symptoms, and who underwent a thyroid ultrasound and a fine needle aspiration with complete medical records. Data were collected from 143 patients diagnosed with thyroid nodules. Of these, 99 patients underwent an ultrasound-guided FNA (Figure 1).

### Study protocol

#### Demographic evaluation

For each patient, we collected the following data: age, sex, associated pathologies, circumstances of nodule discovery, dysthyroid symptoms, and personal or family history of thyroid nodule or carcinoma.

#### Biological evaluation

After detecting a thyroid nodule, the thyroid stimulating hormone (TSH) and serum-free tetraiodothyronine (FT4) were measured using a radioimmunometric assay. Anti-thyroperoxidase antibodies, anti-thyroglobulin antibodies, and calcitonin were measured using an automated immunochemiluminescent assay.

#### Sonographic evaluation

Thyroid ultrasound is the standard imaging modality for thyroid nodules. It enables the establishment of a diagnosis and description of their features. Thyroid ultrasonography was performed by an experienced practitioner using a HITACHI ARIETTA V70 machine. We recorded the ultrasound features of the thyroid nodules such as size (measured in three dimensions: length, width, and height), composition (solid, cystic proportion, or spongiform), echogenicity compared to the surrounding strap muscle or thyroid parenchyma (hyperechoic, isoechoic, hypoechoic, or mixed), nodules margins, presence and type of calcifications, shape (if taller than wide), vascularity, and lymph node.

Thyroid imaging reporting and data systems by the European Thyroid Association (EU-TIRADS) were used to provide a score to stratify the thyroid nodules and select them for fine needle aspiration. EU-TIRADS includes five categories [4]:

- EU-TIRADS 1: Normal thyroid gland
- EU-TIRADS 2: Benign
- EU-TIRADS 3: Low risk of malignancy
- EU-TIRADS 4: Intermediate risk of malignancy
- EU-TIRADS 5: High risk of malignancy.

EU-TIRADS 1 and 2 were excluded since there were no indications of fine needle aspiration.

#### FNA cytology procedure and evaluation

FNA was performed by an experienced senior endocrinologist. Patients were placed in a supine position with a slightly extended neck. Once the lesion was localized, the neck was prepared in a sterile, draped environment. A 25-gauge needle mounted on a 5-ml syringe with support was positioned just above the transducer. The cytological diagnosis was made by an experienced pathologist. The patient should avoid swallowing, talking, or moving during the procedure. It is recommended that the aspirates be taken sequentially from the peripheral areas and from different parts of the nodule to ensure representative sampling [7].

Results were analyzed according to the 2017 Bethesda System for Reporting Thyroid Cytopathology (TBSRTC), which classifies thyroid nodules into six categories [8] :

- Bethesda I: Non-diagnostic or unsatisfactory
- Bethesda II: Benign
- Bethesda III: Atypia of undetermined significance or follicular lesion of undetermined significance
- Bethesda IV: Follicular neoplasm or suspicious for a follicular neoplasm
- Bethesda V: Suspicious for malignancy
- Bethesda VI: Malignant.

For US-guided FNA, the criteria included nodules [4,9]:

- ≥ 1cm classified EU-TIRADS 5
- ≥ 1.5 cm classified EU-TIRADS 4
- ≥ 2cm classified EU-TIRADS 3
- If there were suspicious lymph nodes or extra-thyroidal expansion for thyroid nodules ≤ 1cm

Additionally, FNA was indicated under the following specialized conditions:

- A suspected lymph node or distant metastasis
- High-risk features: Size increase, juxta-capsular nodule (≤ 2mm), superior polar nodule, multifocality suspicion, and age under 40 years.

In our study, cases reported as 'suspicious' by cytology were included in the malignant category for statistical analysis, as both suspicious and confirmed lead to surgical management. Vice versa, cases represented in the benign category include only thyroid nodules with a BETHESDA II classification.

### Outcomes

The primary outcome of our study was to describe the various ultrasound parameters predictive of malignancy (size, echogenicity, microcalcifications, vascularization, and presence or absence of lymph nodes). The secondary outcome was to correlate US features and the clinical and biological characteristics of patients with thyroid nodules with the results of FNA.

### Statistical analysis

The collected data were analyzed using SPSS IBM Statistics, version 21. The results were presented as frequencies and percentages, given that all variables were categorical. In order to assess the association between ultrasound features and the risk of malignancy, the chi-square test and Fisher's exact test were employed. Variables significantly associated with the risk of malignancy were then selected for univariate analysis using a logistic regression model (crude odds ratios). A *P* value < 0.05 was considered statistically significant. Multivariate analysis via a logistic regression model was not employed due to the limited number of malignant nodules. Additionally, sensitivity, specificity, and positive and negative predictive values were calculated to evaluate diagnostic performance.

## RESULTS

**Figure 2:**
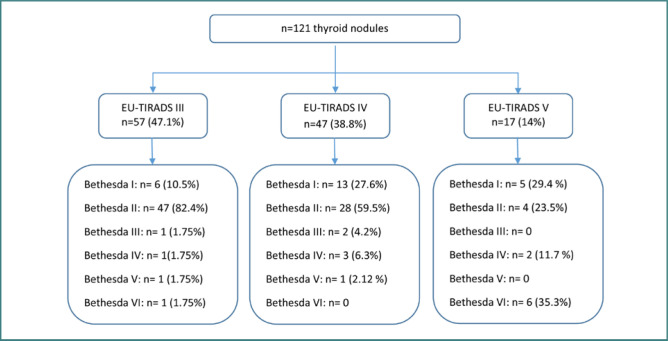
Flow chart showing the distribution of thyroid nodules according to EU-TIRADS score on thyroid ultrasound and corresponding Bethesda classification after FNA

We collected the data from 143 patients with thyroid nodules, of whom 99 underwent an ultrasound-guided FNA, i.e., in 69% of the cases, FNA was performed on 121 nodules in 99 patients (Figure 1). Cytopathology found 79 benign nodules (65.3%), 24 were inadequate for cytological diagnosis (19.8%), seven nodules were malignant (5.8%), six were suspicious for follicular neoplasm (5%), three were indeterminate (2.5%), and two were suspicious for malignancy (1.7%). Figure 2 illustrates the distribution of the 121 thyroid nodules according to the EU-TIRADS score and their corresponding Bethesda classification after the FNA procedure.

To compare the clinical, biological, and US features of malignant and benign nodules, we singled out Bethesda II nodules (benign nodules) and compared them to Bethesda V and VI nodules (Table 1). The general characteristics of the 73 patients with these selected nodules and those who underwent FNA are detailed in Table 1. The age of the patients varied between 10 and 79 years, with an average age of 45.05 ± 14.34 and a female predominance (87.9%). The highest number of cases was seen in the age group ≥ 45 years (54.5%).

**Table 1:** Comparative characteristics of patients undergoing FNA (overall sample and subgroups with benign and malignant nodules)

Variables	All patients *n* = 99 (121 nodules)	Patients with benign or malignant nodules on cytology *n* = 73 (88 nodules)		*P* value*
		Benign nodules (*n* = 65)	Malignant nodules (*n* = 8)	
**Age (years)** **<45 years** **≥ 45 years** **Mean ±SD** **Range**	45 (45.5%) 54 (54.5%) 45.05 ±14.34 10-79 years	30 (46.1%) 35 (53.9%) 44.12 ±13.98 10-79 years	4 (50%) 4 (50%) 46.62 ±16.37 27-75 years	1
**Gender** **Women** **Men**	87 (87.9%) 12 (12.1%)	57 (87.7%) 8 (12.3%)	6 (75%) 2 (25%)	0.300
**Solitary nodules** **Multiples nodules**	59 (60.2%) 39 (39.8%)	41 (64.1%) 23 (35.9%)	2 (25 %) 6 (75 %)	0.054
**Self-examination** **Thyroid symptoms** **Clinical examination** **Incidentally discovered**	36 (37.5%) 25 (26%) 11 (11.5%) 24 (25%)	23 (36.5%) 14 (12.2%) 7 (11.1%) 19 (30.2 %)	3 (37.5%) 4 (50%) 1 (12.5%) 0 (0%)	0.159
**Thyroid function** **Euthyroid** **Hyperthyroid** **Hypothyroid**	84 (84.9%) 2 (2%) 13 (13.1%)	58 (89.2%) 1 (1.5%) 6 (9.2%)	5 (62.5%) 1 (12.5%) 2 (25%)	0.078
**Calcitonin** **Positive** **Negative**	2 (2.1%) 95 (97.9%)	0 (0%) 63 (100%)	1 (12.5%) 7 (87.5%)	0.113
**Anti-TPO Ab** **Positive** **Negative**	6 (6.1%) 92 (93.9%)	3 (4.7%) 61 (95.3%)	1 (12.5%) 7 (87.5%)	0.382

Anti-TPO Ab, Anti Thyroid Peroxidase Antibody

* significance level from the chi-square test comparing characteristics between patients with benign and malignant nodules

In our series, self-palpation was the most common circumstance of discovery (37.5% of cases). Most patients (84.9%) did not exhibit thyroid abnormalities. The levels of calcitonin were mildly elevated in two patients, and thyroid peroxidase antibodies (Anti-TPO Ab) were positive in 6% of cases. Nodules were mostly solitary in 60.2% of cases and multiple in 39.8%.

The ultrasound characteristics of the 121 nodules evaluated in this study are detailed in Table 2, together with the description of the nodules according to the Bethesda classification. The average size of the nodules was 3.04 ± 1.58 cm. Hypoechogenicity was found in 37.2% of nodules. Most nodules (92.6%) had well-defined margins, and the majority (91.7%) had regular contours. Among the nodules with irregular contours (*n* = 10), 20% were classified as benign and 30% as malignant. The presence of microcalcifications was noted in 4.1% of the nodules. Regarding vascularization, it was predominantly peripheral, occurring in 65.83% of all nodules and 67.5% of those classified as benign (Table 2).

**Table 2:** Ultrasound findings according to the FNAC results (n = 121)

Parameters	All nodules *n* = 121	Bethesda I *n* = 24	Bethesda II *n* = 79	Bethesda III *n* = 3	Bethesda IV *n* = 6	Bethesda V *n* = 2	Bethesda VI *n* = 7	*P* value
**Nodules size** **<2cm** **2-4cm** **>4 cm** **Average size**	36 (29.7%) 52 (43%) 33 (27.3%) 3.04±1.58	14 (58.3%) 6 (25%) 4 (16.7%)	13 (16.5%) 42 (53.2%) 24 (30.4%)	2 (66.7%) 1 (33.3%) 0 (0%)	1 (16.7%) 2 (33.3%) 3 (50%)	1 (50%) 0 (0%) 1 (50%)	5 (71.4%) 1 (14.3%) 1 (14.3%)	0.000
**Taller-than-wide¹** **Yes** **No**	12 (9.9%) 109 (90.1%)	3 (12.5%) 21 (87.5%)	5 (6.3 %) 74 (93.7%)	0 (0%) 3 (100%)	1 (16.7%) 5 (83.3%)	0 (0%) 3 (100%)	3 (50%) 3 (50%)	0.049
**Hypoechoic** **Yes** **No**	45 (37.2%) 76 (62.2%)	15 (62.5%) 9 (37.5%)	19 (24.1%) 60 (75.9%)	1 (33.3%) 2 (66.7%)	3 (50%) 3 (50%)	1 (50%) 1 (50%)	6 (85.7%) 1 (14.3%)	0.000
**Margin** **Well-defined** **Ill-defined**	112 (92.6%) 09 (7.4%)	21 (87.5%) 3 (12.5%)	76 (96.2 %) 3 (3.8 %)	3 (100%) 0 (0%)	4 (66.7 %) 2 (33.3%)	2 (100 %) 0 (0%)	6 (85.7%) 1 (14.3 %)	0.071
**Irregular borders** **Yes** **No**	10 (8.3%) 111 (91.7%)	3 (12.5 %) 21 (87.5 %)	2 (2.5%) 77 (97.5 %)	0 (0%) 3 (100 %)	2 (33.3%) 4 (66.7%)	0 (0%) 2 (100 %)	3 (42.9%) 4 (57.1 %)	0.003
**Microcalcifications** **Yes** **No**	5 (4.1%) 115 (95.8%)	0 (0) 24 (100%)	3 (3.8 %) 76 (96.2 %)	0 (0%) 3 (100 %)	1 (16.7 %) 5 (83.3%)	0 (0%) 2 (100 %)	2 (28.6%) 5 (71.4%)	0.062
**Vascularity** **None** **Peripheral** **Central** **Mixed**	12 (10%) 79 (65.83%) 4 (3.33%) 25 (20.83%)	7 (12.5 %) 17 (79.2 %) 0 (0%) 2 (8.3%)	5 (6.5 %) 52 (67.5 %) 1 (1.3 %) 19 (24.7%)	0 (0%) 3 (100%) 0 (0%) 0 (0%)	0 (0%) 5 (83.3%) 0 (0%) 1 (16.7%)	0 (0%) 1 (50%) 0 (0%) 1 (50%)	0 (0%) 1 (16.7%) 3 (50%) 2 (33.3%)	0.015
**EU-TIRADS ^2^** **III** **IV** **V**	57 (47.1%) 47 (38.8%) 17 (14.1%)	6 (25%) 13 (54.2%) 5 (20.8%)	47 (59.5 %) 28 (35.4%) 4 (5.1%)	1 (33.3%) 2 (66.7%) 0 (0%)	1 (16.7%) 3 (50%) 2 (33.3%)	1 (50%) 1 (50%) 0 (0%)	1 (14.3%) 0 (0%) 6 (85.7%)	0.000

¹ A taller-than-wide thyroid nodule is characterized by an anteroposterior diameter greater than its transverse diameter

^2^ EU-TIRADS: European Thyroid Imaging Reporting and Data System

Concerning the EU-TIRADS classification, most of the benign nodules were classified as EU-TIRADS III (59.5%), whereas 85.7% of malignant nodules were classified as EU-TIRADS V (Table 2).

Of 121 thyroid nodules that underwent FNA, 88 were classified as benign (Bethesda II) or malignant (Bethesda V and VI). Table 3 summarizes the US findings of each group and presents the factors associated with malignancy. The results of our statistical analysis revealed a correlation between specific critical ultrasound features and the risk of malignancy. These findings are presented in Table 4, while Table 5 provides a detailed summary of the performance of various ultrasound features.

**Table 3:** Ultrasound characteristics associated with malignancy in thyroid nodules (n = 88)

Parameters	Benign *n* = 79 (%)	Malignant n = 9 (%)	*P*
**Nodules size** **<2cm** **2-4cm** **>4 cm** **Mean±SD**	13 (16.4 %) 42 (53.2 %) 24 (30.4 %) 3.3±1.5 cm	6 (66.7%) 1 (11.1 %) 2 (22.2%) 2.7±1.6 cm	0.003
**Taller-than-wide¹** **Yes** **No**	5 (6.3%) 74 (93.6%)	3 (37.5%) 5 (62.5%)	0.023
**Hypoechoic** **Yes** **No**	19 (24 %) 60 (76 %)	7 (77.8%) 2 (22.2%)	0.002
**Marked hypoechogenicity** **No** **Yes**	17 (94.4%) 1 (5.6%)	5 (71.4%) 2 (28.6%)	0.006
**Ill-defined margins** **No** **Yes**	76 (96.2%) 3 (3.8 %)	8 (88.8%) 1 (11.2%)	0.356
**Irregular borders** **Yes** **No**	2 (2.5%) 77 (97.5%)	3 (33.4%) 6 (66.6%)	0.007
**Microcalcifications** **Yes** **No**	3 (3.8%) 76 (96.2%)	2 (22.2%) 7 (77.8%)	0.080
**Vascularity** **None and peripheral** **Central and mixed** Central Mixed	57 (74%) 20 (26%) 1(1.3%) 19 (24.7%)	2 (25%) 6 (75%) 3 (37.5%) 3 (37.5%)	0.009
**EU-TIRADS^2^** **III** **IV** **V**	47 (59.5%) 28 (35.4%) 4 (5.1%)	2 (22.2%) 1 (11.1 %) 6 (66.7%)	0.000

¹A taller-than-wide thyroid nodule is characterized by an anteroposterior diameter greater than its transverse diameter

^2^EU-TIRADS: European Thyroid Imaging Reporting and Data System.

**Table 4:** Ultrasound features of thyroid nodules according to the risk of malignancy

Parameters	Univariate analysis	
	OR* (95% Cl**)	*P* value
**Nodules size** **>4 cm (Ref)** **2–4 cm** **<2 cm**	1 (Reference) 5.53 (0.97–31.45) 0.28 (0.025–3.31)	0.013 0.53 0.317
**Taller-than-wide¹** **Yes** **No**	8.88 (1.62–48.30)	0.012
**Hypoechoic** **Yes** **No**	11.05 (2.11–57.78)	0.004
**Marked hypoechogenicity** **No** **Yes**	0.147 (0.011–1.979)	0.148
**Vascularity^2^** **None and peripheral** **Central and mixed**	8.55 (1.59–45.8)	0.012
**Irregular borders** **Yes** **No**	19.25 (2.67–138.4)	0.003
**EU-TIRADS^3^** **III** **IV** **V**	1 (Reference) 0.839(0.073–9.68) 35.25(5.28–23.24)	0.000 0.888 0.000

¹A taller-than-wide thyroid nodule characterized by an anteroposterior diameter greater than its transverse diameter

^2^Vascularity: devised in two groups: none and peripheral/central and mixed

^3^EU-TIRADS: European Thyroid Imaging Reporting and Data System

* OR: Odds Ratio, **CI: Confidence Interval

**Table 5:** Diagnostic performance of ultrasound features in thyroid nodule assessment

	Sensitivity (%)	Specificity (%)	PPV (%)	NPV (%)
**Nodules size** **(< 2cm)**	**66.7**	**82.2**	**30**	**95**
**Taller-than-wide¹**	**37.5**	**93.7**	**37.5**	**93.6**
**Hypoechoic**	**77.8**	**75.9**	**26.9**	**96.7**
**Irregular borders**	**33.3**	**97.5**	**16**	**93.5**
**Vascularity^2^**	**75**	**74**	**23**	**96**

¹A taller-than-wide thyroid nodule is defined by having an anteroposterior diameter greater than its transverse diameter

^2^Vascularity: devised in two groups: the first one; None and Peripheral / the second; Central and mixed

The mean size of malignant nodules was 2.7 ± 1.6 cm, while the majority had a size less than 2 cm (with a sensitivity of 66.7%). In contrast, benign nodules typically ranged from 2 to 4 cm, comprising 53.2% of this group). Statistical analysis revealed a significant association between nodule size and malignancy risk (*P* = 0.003). Additionally, only eight nodules (9.2%) were taller-than-wide, of which 3 (37.5%) were malignant (*P* = 0.023) with a specificity of 93.7% and a sensitivity of 37.5%. Most of the malignant nodules were hypoechoic (77.8%) (*P* = 0.002), whereas 76% of the benign nodules were not hypoechoic, the specificity and sensitivity values being 75.9% and 77.8%, respectively. The positive and negative predictive values for hypoechoic nodules in predicting malignancy were 26.9% and 96.7%, respectively. Well-defined margins were observed in 96.2% of the benign nodules, and the ill-defined margin was not associated with malignancy (*P* = 0.356). However, irregular margins were significantly associated with an increased risk of malignancy (*P* = 0.007), with a high specificity of 97.5% and a sensitivity of 33.3%. Microcalcifications were noted in 28.6% of malignant nodules and were not significantly associated with malignancy (*P* = 0.080). Color Doppler imaging revealed central and mixed vascularization in 75% of the malignant nodules (*P* = 0.009) with a negative predictive value of 96% (Tables 3-5).

## DISCUSSION

In our study, out of all 121 thyroid nodules, 65.5% were benign. There were no statistically significant differences between the two categories (benign and malignant) regarding sex, age, thyroid function, and solitary or multiple nodules (*P* > 0.05). The risk of malignancy was determined by the presence of hypoechoic echogenicity, vascularity, nodule size, irregular border, and shape (diameter taller than wide).

The mean age of 45 years observed in our study aligns with findings from other research [10,11], although we found no significant association between age and the risk of malignancy. Contrasting evidence from the literature shows that the risk of malignancy increases in individuals aged 45 years and older in some studies [12], while other reports suggest a higher risk in younger individuals under 45 years [13]. Our study included approximately 87.9% of women, which is in line with previous studies that demonstrate a higher prevalence of thyroid nodules in women [14]. Women seem more prone to developing thyroid nodules, while men are more likely to develop a malignant nodule [10,15]. Nevertheless, our study did not show significant differences in malignancy rates between genders, which could be attributed to the relatively smaller sample size compared to other studies. The initial evaluation of patients with thyroid nodules typically includes measuring TSH levels. Globert *et al*. [16] reported that higher serum TSH levels are associated with an increased risk of thyroid cancer in patients with nodules. However, in the current study, TSH levels were not associated with malignancy.

Although nodule size is routinely measured, its impact on the risk of thyroid cancer is still under discussion. Some studies highlight that the size of the nodule plays an important role in determining the risk of malignancy [17], while others find no significant link between nodule size (greater than 4 cm) and malignancy risk [18-20]. In our study, 66% of malignant nodules were less than 2 cm in diameter. When combined with other malignant features, nodule size may be a useful predictor of malignancy.

Studies by Boelaert *et al*. [21] and Kim *et al*. [22] suggest that single nodules are more likely to be malignant, contrary to other research, which argues that multiple nodules carry a higher risk [12]. The present study found no significant correlation between nodule multiplicity and malignancy risk.

Several recent studies have shown that a round appearance, a taller-than-wide (TTW), or round nodules can suggest malignancy [23,24]. Ren J. *et al*. [25] found that TTW > 1, along with other ultrasound risk factors, has great diagnostic performance for the diagnosis of papillary thyroid carcinomas, especially in small-sized nodules, with high specificity (96.8%) and sensitivity (81.4%). In our study, this characteristic was observed in 37.5% of malignant nodules (*P* = 0.012).

Consistent with literature [14,26], hypoechogenicity is commonly observed in malignant thyroid nodules. Our study reinforces this association, showing that 66.7% of severely hypoechoic nodules were malignant (*P* = 0.148). Our observations regarding irregular borders as an indicator of malignancy were consistent with those reported in the literature [9].

Intranodular vascularity appeared to be associated with malignant lesions, which is consistent with the findings of Lyshchik *et al*. [27]. This association was statistically significant in the current study (*P* = 0.012). Some reports suggest that Doppler color does not aid in differentiating between malignant and benign nodules [28, 29]. In addition, the presence of microcalcifications is a well-known risk factor for malignancy in thyroid nodules, as identified in a comprehensive meta-analysis conducted by Campanella *et al*. [30]. This finding was not confirmed in our study.

The study results highlight the diagnostic value of thyroid US and the importance of the EU-TIRADS score in characterizing thyroid nodules and indicating FNA. Although the EU-TIRADS III score was more common in benign nodules, our study found one patient with EU-TIRADS III who had a BETHESDA VI cytology result, indicating the need to consider the risk of malignancy with EU-TIRADS III.

FNA is a crucial tool in guiding thyroid nodule management and is considered the gold standard for determining whether nodules are benign or malignant [31]. It is recommended that a second FNA be performed on nodules classified as Bethesda I and III. Generally, the percentage of thyroid cytology classified as Bethesda III should not exceed 7 to 10%, while Bethesda I cytology rates are ideal at less than 10% [32, 33]. In our study, these percentages were 2.5% and up to 19%, respectively.

The disparities in ultrasound features between our study and others can be attributed to differences in inclusion and exclusion criteria and the basis for distributing control groups.

Our study's strengths lie in being the first of its kind conducted in our country. Our study highlights the significance of adhering to the guidelines for managing thyroid nodules and how the use of ultrasound and FNA in combination can enhance the diagnostic accuracy of thyroid nodules.

Our study has several limitations. Its retrospective design restricted our ability to assess some factors comprehensively, which may have influenced the results. Additionally, some clinical information was missing from the medical records during data collection, which could have contributed to gaps in data analysis. Additionally, the sample size of our study was relatively small, and the number of malignant thyroid nodules was also limited. This has a significant impact on the accuracy of the other statistical analyses.

## CONCLUSION

The identification of benign and malignant thyroid nodules is a crucial aspect of clinical practice. Our findings confirm that the EU-TIRADS score and the fine needle aspiration remain valuable and straightforward methods for assessing the risk of malignancy in thyroid nodules, determining patients who require surgery, and avoiding unnecessary procedures.

## Personal thanks

The authors want to acknowledge Dr Siham Benouda, Dr Amine Bouazzaoui, and Dr Asmae Lekfif.
